# Tear Osmolarity in the Diagnosis of Systemic Dehydration and Dry Eye Disease

**DOI:** 10.3390/diagnostics11030387

**Published:** 2021-02-25

**Authors:** Anthony J. Bron, Catherine Willshire

**Affiliations:** 1 Nuffield Department of Clinical Neurosciences and Nuffield Laboratory of Ophthalmology, University of Oxford, Oxford OX2 6HZ, UK; 2 Ophthalmology Research, Hinchingbrooke Hospital, North West Anglia Trust, Huntingdon PE29 6NT, UK; catherine.willshire@nhs.net

**Keywords:** basal tear osmolarity, dehydration, dry eye, plasma osmolality, tonicity, hyperosmolar

## Abstract

Systemic dehydration due to inadequate water intake or excessive water loss, is common in the elderly and results in a high morbidity and significant mortality. Diagnosis is often overlooked and there is a need for a simple, bedside diagnostic test in at-risk populations. Body hydration is highly regulated with plasma osmolality (pOsm) being tightly controlled over a wide range of physiological conditions. By contrast, normal tear osmolarity (tOsm) is more variable since the tear film is exposed to evaporation from the open eye. While plasma hyperosmolality is a diagnostic feature of systemic dehydration, tear hyperosmolality, with other clinical features, is diagnostic of dry eye. Studies in young adults subjected to exercise and water-deprivation, have shown that tOsm may provide an index of pOsm, with the inference that it may provide a simple measure to diagnose systemic dehydration. However, since the prevalence of both dry eye and systemic dehydration increases with age, the finding of a raised tOsm in the elderly could imply the presence of either condition. This diagnostic difficulty can be overcome by measuring tear osmolality after a period of evaporative suppression (e.g., a 45 min period of lid closure) which drives tOsm osmolality down to a basal level, close to that of the pOsm. The arguments supporting the use of this basal tear osmolarity (BTO) in the diagnosis of systemic dehydration are reviewed here. Further studies are needed to confirm that the BTO can act as a surrogate for pOsm in both normally hydrated subjects and in patients with systemic dehydration and to determine the minimum period of lid closure required for a simple, “point-of-care” test.

## 1. Introduction

Water-loss dehydration, due to a net loss of hypotonic body fluids [1,2,3,4], is a common condition in the elderly, responsible for functional disability, poor health outcomes, and death [3,5] on a global scale. In the UK, dehydration affects twenty per cent of older people living in residential care [6] and is responsible for unplanned hospital visits [7] with 40% found to be dehydrated on hospital admission [8]. In the US NHANES study [9] dehydration was identified in over 60% of a large cohort of elderly people living in the community, with preclinical dehydration observed in 40% and a further 20% showing current dehydration.

Optimal body water content (“euhydration”), is characterised by a plasma osmolality (pOsm) in the region of 285–295 mOsm/kg [10] (see later). In “systemic dehydration”, due to inadequate water intake or excessive water loss, the pOsm rises above this level. Mild dehydration may be relatively symptom free and overlooked in the community when the facilities to determine the pOsm in blood samples are not available. There is a need for simple bedside tests to detect it in the community setting.

One possible approach is to measure tear osmolarity (tOsm). It has been shown that when tear evaporation is prevented by eye closure, the tOsm is driven down to a basal level that is close to that reported for the plasma [11,12,13]. This is assumed to be the lowest tOsm level that can be achieved in resting eye conditions and we have termed this value the Basal Tear Osmolarity (BTO). We have proposed it as a metric in the diagnosis of systemic dehydration. Since pOsm is normally controlled within narrow limits, it was predicted that the BTO would similarly have a small variance.

Dry eye disease (DED) is characterised by tear hyperosmolarity and its severity is usually gauged by comparing a patient’s tOsm, measured casually or in clinic conditions, with population tOsm norms [14]. We have suggested that a comparison of a patient’s tOsm with their baseline BTO would be a better gauge of tear hyperosmolarity and therefore of DED severity.

In the following account we summarise the evidence that supports the potential value of the BTO as a new metric. Fuller details of the personal research cited here are published elsewhere [11,12,13]. 

## 2. Terminology

The terms osmolarity, osmolality, and tonicity refer to the concentration of particles dissolved in a solution. Their use is critical to a discussion of systemic dehydration and DED. The “osmolarity” of a solution is the number of osmoles per litre of solvent, usually expressed as milliosmoles. This is the preferred term when referring to the concentration of solutes in the tears [15]. The term *osmolality* refers to the number of millisomoles *per kilogram* of solution and is the term applied when direct measurement is made on the plasma by vapour pressure or depression of freezing point osmometry. Because the opportunity to carry out osmometry in clinical practice is limited, it is more usual to calculate the *osmolarity* of a serum sample rather than its *osmolality*, based on the concentration of selected blood constituents such as Na^+^, K^+^, Cl^−^, urea, and glucose. Osmolarity is influenced by temperature and pressure whereas osmolality is not. Clinically the numerical difference between the two terms is negligible but the formula selected to make the calculation is of importance [16]. Here we use either term according to its literature source.

The tonicity of an aqueous solution is the number of osmotically effective particles dissolved in the solution [17]. An osmotically effective particle diffuses poorly across a semi-permeable membrane and when its concentration on one side of a membrane is greater than on the other, it will draw water across the membrane along the concentration gradient. A formula developed to estimate plasma or serum tonicity (or effective osmolarity or osmolality) should not include those solutes, such as urea, that diffuse readily across cell membranes and cannot exert an osmotic effect [2]. The formula used has a bearing on comparisons made between the osmolarity of the tears measured by the TearLab^®^ device and that estimated for the serum or plasma.

## 3. The Tears

### 3.1. Introduction

**Figure 1:**
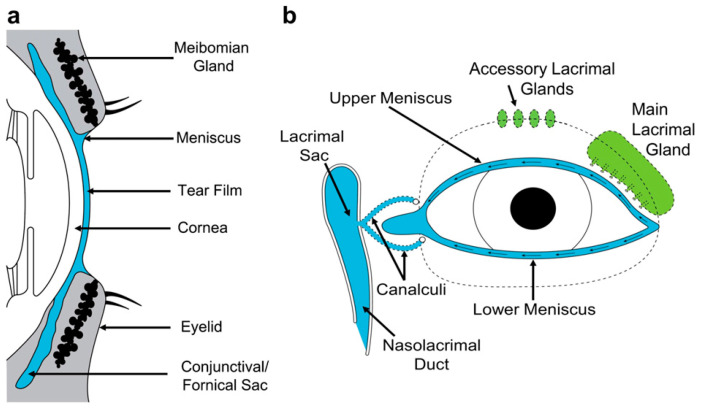
(**a**) Sagittal view of the eye to show tear distribution. (**b**) Schematic view of the lacrimal drainage system seen *en face*. (Reprinted with permission from ref. [18]. Copyright 2009 Elsevier Ltd.)

The tears comprise an aqueous solution that bathes, moistens and protects the exposed conjunctiva and cornea and the mucosal posterior margins of the lids of the open eye [18]. With the eyes open, the tears occupy three compartments (Figure 1). The tear menisci and tear film are two compartments in continuity. Together they make up the *pre-ocular fluid* that overlies that part of the open eye that is exposed to evaporative water loss. The third compartment, consisting of the tears residing in the retro-tarsal spaces and fornices, is not thus exposed. 

Aqueous tears are secreted into the supero-lateral fornix by the *main and palpebral parts* of the lacrimal gland and into the upper fornix by the *accessory* lacrimal glands, with a smaller contribution from the conjunctiva and probably, cornea. Fluid is drained via the nasolacrimal system and lost by evaporation. The tear film is refreshed by blinking, at a rate of between 10 and 30 per minute [19]. During blinking there is mixing of fluids between the menisci and the fornical compartments [20]. Between blinks it is likely that fluid flows between the compartments, amplified by eye movements [21].

Tears are actively secreted by the lacrimal gland, which is their major source [22,23,24,25,26,27]. However, since dry eye does not necessarily develop after dacryoadenectomy (removal of the main and palpebral parts of the gland) [28,29,30,31] and the Schirmer response may remain normal after palpebral dacryoadenectomy (which removes the secretory component from both the main and palpebral parts of the gland) [28,30], it is recognised that there is an additional contribution from the accessory lacrimal glands, the conjunctival epithelium [25,32,33,34] and to a lesser extent the corneal, epithelium [35]. Cerretani and Radke [36] estimated that an osmotically-determined flow across the conjunctiva and cornea could account for up to 10% of the total. 

A general view of tear flow regulation is that when the eyes are open, the temperature, humidity, and airflow equable and the subject free from physical or emotional stress, tear flow is low, but sufficient to maintain a moist ocular surface at all times [37]. The lacrimal component increases substantially during emotional tearing and in response to intense light or a corneal foreign body [33,38,39]. An early concept of tear secretion was that there were *basic* and *reflex* tear secretors [40], with the basic secretors, (accessory lacrimal glands and the glands of Kraus), considered to be non-innervated and responsible for a steady, low level of tear secretion, while the reflex secretors, the innervated main and palpebral lacrimal glands, were the basis of responses to reflex and emotional stimuli. It has since been shown that the accessory lacrimal glands receive an identical afferent and efferent innervation to that of the main and palpebral glands [41], so that a differentiation of that kind is not acceptable. 

Jordan and Baum [37] concluded on the basis of fluorophotometric studies, that the rate of tear secretion depended on the size of psychogenic and reflex sensory inputs to the lacrimal system. Measured tear flow was greatly reduced by dense topical anaesthesia and as they pointed out, “the abolition of all sensory input and elements of supranuclear and psychogenic stimulation under general anaesthesia [42], resulted in a tear flow rate approximating zero.” In keeping with the expectation that tear flow and volume would be reduced during sleep Gilbard et al. [43] found that immediately upon lid opening, after overnight sleep, no inferior tear meniscus was visible, but that it appeared at the first blink. In that study the tOsm immediately on waking, at 6.00 a.m., was significantly lower than that measured at 9.00 a.m. There was no difference between tOsms measured in the *meniscus and lower fornix* at 6.00 a.m., but the meniscus tOsm was significantly higher than that in the fornix at 9.00 a.m. and 9.00 p.m. (*p* < 0.0005 and *p* < 0.0005, respectively). 

### 3.2. The Lacrimal Secretion

The secretory cells of the lacrimal gland make up about 80% and its duct cells 10–12% of its glandular mass [33] while the accessory glands account for about a tenth [44].

The *lacrimal secretion* is modified as it passes through the lacrimal ducts by the addition of water and electrolytes, particularly K^+^ and Cl^−^ ions [22,25,45,46,47], so that its composition differs from that of the *lacrimal fluid* delivered into the conjunctival sac. 

### 3.3. Tear Mixing and Distribution

As noted, the lacrimal, conjunctival, and corneal fluids are mixed and distributed by tear flow, blinking [18] and to a lesser extent eye movements [21] and it is this composite fluid that is called *the tears* and assayed from meniscus samples.

### 3.4. Tear Osmolarity

Tear osmolarity is determined by the interaction of tear production, evaporation and drainage and of diffusion across the epithelia of the conjunctival sac, on its composition. It is measured on samples drawn from the lower tear meniscus in resting conditions. The subject is allowed to adapt to the ambient conditions of a draft-free room whose temperature and humidity are regulated [13,48]. In the studies cited below, the temperature selected was 23 °C and relative humidity (RH) was 45%, based on recommendations for equable ambient conditions in the workplace [49]. In clinical practice these environmental conditions may not be regulated, standardised and documented or even mentioned in published reports, so that uncharted differences in evaporative loss may occur. The term *casual* tOsm may be used to refer to values acquired outside of the clinic.

In early studies of tOsm there were technological factors that influenced the accuracy of measurements. Early methods [50] such as *vapourpressure* (VP) osmometry required relatively large tear volumes (5 µL) and prolonged collection times inevitably led to eye irritation, reflex tearing and altered tear composition. Normal tear volume is in the order of 7 µL [19] and the volume is further reduced in aqueous-deficient dry eye. Reflex tearing results in lower tOsm values [51,52,53]. Additionally, evaporation from the sample during processing could influence the analysis [54]. A later adaptation to the Wescor osmometer (Wescor 5520) permitted accurate analysis of down to 0.8 µL [55].

The problem of sample volume was partially resolved by the introduction of the *depression-of-freezingpoint* (DFP) osmometer to measure tOsm. Osmolality depends on the total number of dissolved particles in a solution and is directly proportional to its freezing point. The Clifton nanolitre osmometer required a sample of 0.5–2 µL [53,55,56,57], which could be collected with limited stimulation of reflex tears. The sample could then be sealed in its collecting tube for transport and later measurement in the laboratory. It was used extensively to establish the role of tear hyperosmolarity in inducing dry eye disease [51,56,57]. However, although this technique was highly accurate it was labour-intensive and technically demanding, with some risk of evaporative loss during handling, making its use impractical in a general clinic setting. 

An effective answer to the problem came with the development of the TearLab^®^ Osmolarity System [14,58,59,60], which uses the principle of temperature-compensated electrical impedance to estimate osmolarity. The electrical conductivity of a solution depends on the number of charged particles present, such as ions, which represent the bulk of the dissolved solute in the tears. For this reason, the derived tOsm is close to the true osmolarity of the tears. The TearLab^®^ device requires the smallest sample of all the osmometers (50 nL). Tear fluid is taken up rapidly by the microfluidic system without risk of evaporative loss and because there is limited or no contact with the ocular surface, the risk of stimulating reflex tearing is small. Within the system there is an almost instantaneous analysis of the electrical properties of the tears, which are then converted into an osmolarity value. The precision and accuracy of the TearLab^®^ osmometer compares well with that of commercially available, VP and DFP devices, which provide the laboratory gold standards in osmolarity measurement of [59,60,61,62].

A question arises as to the comparability of these devices when measuring osmolality or osmolarity in the plasma or the tears. The VP and DFP osmometers take into account all particles in solution and provide a true record of *osmolality*. However, since such osmometers are not routinely in clinical use, it is common to derive *serum osmolarity or osmolality* from its chemical composition. There is some debate as to the best equation to use. In an evaluation of 38 predictive equations, the best equation identified by Siervo et al., [6] predicted serum osmolality to within 2%, in >80% of participants, regardless of diabetes or hydration status and had a sensitivity and specificity for impending dehydration (≥295 mmol/kg) of 79% and 89% and for current dehydration (>300 mmol/kg) of 69% and 93%, respectively. Like other available equations, this one (osmolarity = 1.86 × (Na^+^ + K^+^) + 1.15 × glucose + urea + 14; all in mmol/L) [63], contains a component for urea, an uncharged molecule that is not detected by the TearLab^®^ device and therefore is not taken into account in the TearLab^®^ estimate of tear osmolarity. It may be presumed that if the VP, DFP, and TearLab^®^ devices were used to measure the same aqueous solution, containing Na^+^, glucose, and urea, the TearLab^®^ value would be lower by the amount representing the urea present since the VP and DFP techniques respond to the presence of all dissolved particles. 

Like urea, glucose is an uncharged molecule which is not recognised by the TearLab^®^ device. While the plasma concentration of glucose in non-diabetic subjects is between 60 and 99 mg/dL, equivalent to 3.3–5.5 mOsm/L, its concentration in normal tears is 3.6 mg/dL [64], equivalent to 0.2 mOsm/L and representing a negligible osmotic load. However, this value would be expected to rise in DED, due to a loss of physical barriers associated with ocular surface inflammation [21] and is significantly raised in diabetes mellitus [65]. The concentration of proteins in normal tears is similarly low and their molecular weight high, so that their osmolar contribution can be ignored. 

As noted earlier, tOsm measured with the TearLab^®^ system correlates well with that measured by the Clifton DFP osmometer and it has therefore been possible to use this method, to provide an indirect, but precise measure of tOsm. 

### 3.5. Tear Osmolarity in Normal Eyes, in Open Eye Conditions

Based on a meta-analysis of several studies in adults, using chiefly DFP or VP methods, Tomlinson et al. reported normal tOsm to be 302 ± 9.7 mOsm/L [66]. Similar values have been reported by other researchers, using the TearLab^®^ electrical conductivity device (Table 1).

The effective osmolality to which most tissues of the body are exposed through contact with the interstitial fluid, lies within the narrow band of pOsm, 285–295 mOsm/kg [2,9,10] which is generally below that reported for normal tears, with little overlap, although it is possible that many of the normal studies included “normal” subjects based primarily on symptoms and could have included some mild dry eye patients in the cohort, thereby increasing the average values. The Nolfi study used more stringent parameters on both symptoms and signs for normal inclusion and reported values closer to the upper end of the pOsm range [72]. Clinically, a pOsm of >295 and ≤300 mOsm/kg is designated as “impending” or “preclinical” systemic dehydration, while a value of >300 mOsm/kg corresponds to “current” systemic dehydration.

It will be seen that plasma levels of osmolality which represent preclinical and the lower range of current clinical dehydration (i.e., up to 300 mOsm/L) would be normal for the ocular surface (i.e., 302 ± 9.7). Putting this another way, the lower normal range of tOsm would correspond to that which defines “preclinical dehydration” for the body as a whole and levels of osmolarity within the upper normal range for the tears would correspond to “current systemic dehydration”, threatening some key organs of the body. It seems that, compared to other tissues, the ocular surface, exposed to tears with an osmolarity ranging from 292.3 to 311.7 mOsm/L [66], is protected from any damaging effects of the osmolarity to which it is routinely exposed.

### 3.6. Tear Osmolarity in Dry Eye Disease

Dry eye disease is a symptomatic eye disorder in which evaporative water loss from the exposed ocular surface, results in a damaging tear hyperosmolarity. There are two major forms. *Aqueous-deficient dry eye* (ADDE) is due to reduced lacrimal secretion. It occurs when water evaporates at a normal rate in the presence of a reduced tear flow. In *evaporative dry eye* (EDE), tear hyperosmolarity results from excessive evaporation, in the presence of normal lacrimal secretory function. This is due to dysfunction of the tear film lipid layer, either as a barrier (a role which is currently under debate [73,74] or as a key element in maintaining tear film stability. Tear film break-up in the blink interval amplifies tear hyperosmolarity and additionally, degrades optical performance when tear instability and breakup intrude upon the visual axis [75]. Many hybrid forms of dry eye exist [76].

Tear hyperosmolarity is accepted to be the central mechanism of both ADDE and EDE, responsible for initiating and perpetuating a vicious circle of inflammatory events at the ocular surface in dry eye [77,78,79,80]. Tear osmolarities up to 519 mOsm/L have been reported clinically [56] and higher levels have been predicted at sites of tear film breakup on the basis of modelling [73]. Exposure of human corneal epithelial cells (HCECs) to hyperosmolar stress, with osmolarities ranging between 330 and 512 mOsm/Kg, induces activation of the MAPK signaling pathway, expression of cytokines, IL-1b, TNF-a, IL-8, and IL-6 [81] and of metalloproteases, MMPs-1, -3, -9, and -13 [82], that are clinical biomarkers for inflammatory events at the ocular surface in DED [79]. There is also evidence of a direct cytotoxic effect of hyperosmolarity on HCECs in culture [83].

Tear osmolarity has been proposed as the gold standard in dry eye diagnosis [57,84,85] and the best single measure of DED [86]. 

In a multicentre study the most sensitive threshold distinguishing normal from mild/moderate dry eye disease was 308 mOsm/L and the most specific cut off was 315 mOsm/L [59,84]. At a cutoff of 312 mOsms/L, tear hyperosmolarity exhibited 73% sensitivity and 92% specificity [14]. Sullivan et al., [14] concluded that tear film osmolarity, due to its “linearity, objectivity, quantitative nature, and operator independence” was the single best test to assess disease severity, used in conjunction with clinical assessment. In terms of tear osmolarity, severity is determined by comparison with values from a control population with normal eyes. In the material that we present below, we suggest that a subject’s own basal level (the BTO) would supply a more appropriate comparator.

### 3.7. The Impact of Environment and Behaviour on Tear Osmolarity

When the eyes are open, the osmolarity of the tears is modified by evaporation to an extent that depends on ambient humidity [87,88], air temperature [89] airflow [73], the length of the blink interval, and area of the exposed ocular surface [90,91]). Low relative humidity (RH), high wind speed, raised air temperature, a wide palpebral aperture and an extended blink interval, all increase tear osmolarity [48,75]. The lacrimal secretion is generally considered to be iso-osmotic (and isotonic), or slightly hypo-osmotic [50,56,69] with respect to plasma. The osmolarity of tears sampled from the menisci is considered to be higher than that of secreted tears [36,69,92,93] but lower than that of the tear film, the latter due to the differential effect of evaporation on these compartments during the blink interval [18].

The ionic composition of the tears, determined by the secretory process [22,25,47] differs from that of the plasma. While the concentrations of Na^+^ and HCO_3_^−^ are similar [94,95,96,97], those of K^+^ and Cl^−^ are higher in the tears [98]. There is evidence in the rabbit [22,97,99] and rat [100] it appears that K^+^ and Cl^−^ ions are added to the lacrimal fluid in the lacrimal duct.

### 3.8. The Effect of Tear Flow-Rate on Tear Osmolarity

It is a clinical requirement for tOsm measurements that tears are assessed under resting conditions that avoid stimulation of reflex tears. With the TearLab^®^ device, rapid tear sampling without stimulating symptoms makes this a reasonable expectation and further, the clinician can decide in advance to reject results associated with a prolonged collection time or symptoms of irritation during collection. 

It might be predicted, even in the absence of altered ionic exchanges within the lacrimal gland or the ducts, that an increased tear flow alone, induced reflexly during sampling or as part of an emotional response, would cause a fall in tOsm, because of the diminished impact of evaporation when flow is high. Nelson and Wright found that when six normal subjects were exposed to the beam of a slit lamp for 5 s, to induce reflex tearing, there was a significant, 5%, fall in tOsm, measured by DFP, from 303.2 ± 7.2 mOsm/kg (range 287–312 mOsm/kg) to 289.5 ± 6.8 mos/kg (range 275–298 mos/kg) [39]. Similarly, lacrimal fluid osmolarity has been reported to be inversely proportional to flow rate in the rabbit [101,102].

### 3.9. The diurnal Variation of Tear Osmolarity

**Figure 2:**
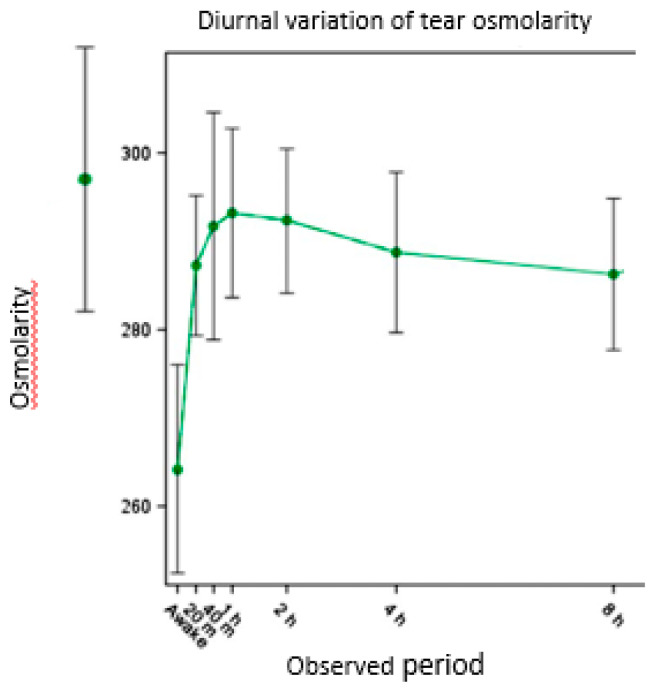
Diurnal tOsm over a 14-h period. A baseline tOsm measurement was made before a period of sleep. Tear osmolarity was then measured on waking and for 8 h after that time. (Adapted with permission from ref. [69]. Copyright 2013 Wolters Kluwer Health, Inc.)

A diurnal variation of tOsm has been reported by several researchers, with the tears being hypo-osmotic on waking [15,50,52,69] and tOsm rising during the day. Niimi et al., [69] using the TearLab^®^ device, measured tOsm at bedtime (taken as base-line) and on waking after 6–8.5 h sleep. Compared with baseline tOsm (297 ± 15 mOsms/L) and tOsm occurring later in the day, tears on waking were found to be significantly hypo-osmotic, at 264 ± 14 mOsms/L. After waking, tOsm rose quickly in the first 10 min, reaching baseline levels within the first 40 min (*p* = 0.085) (Figure 2)

Niimi et al. attributed the tear hypo-osmolarity on waking to the suppression of evaporation by lid closure and possibly to reflex tearing on eye opening [69]. The TearLab^®^ device used by Niimi et al. was modified to allow the recording of readings below the figure of 275 mOm/L, normally the display limit for the instrument [94]. However, given that the normally cited pOsm level is in the region of 285–295 mOm/kg [2,9,10] it is not possible to explain how the low levels on waking occurred. They instructed their subjects to blink three times and to squeeze their eyes shut to release fresh tears prior to tear collection; this may have influenced the outcome but does not explain it. More likely, the piecewise linear calibration curve modification used to estimate values in that region introduced some inaccuracy, as it was unknown prior to the study how low the initial osmolarity upon waking actually was. While the extrapolation of the low end of the calibration is subject to some estimation error, what was clear is that the measured values were significantly below the previously verified limits of 275 mOsm/L for the standard machine.

## 4. Body Hydration and Dehydration

### 4.1. Introduction

Total body water (TBW) makes up about 50–60% of the body mass, with about two thirds being intracellular [103], and blood contributing about 8% [104,105]. Water is lost from the body as insensible perspiration and sweat, respiratory water vapour, urine, and faeces and replaced by fluid intake, including the water in foodstuffs. At sea level, the amount of water lost as respiratory vapour is balanced by metabolic water production [106].

Regulation of water balance is fundamental to survival and is achieved physiologically by a combination of water conservation and water intake stimulated by thirst. Water conservation is achieved by the action of antidiuretic hormone (arginine vasopressin—AVP) on renal water absorption [107] stimulated by an increase in pOsm. Signals from hypothalamic osmoreceptors (e.g., TRPV1) [108,109] signifying changes in cell volume [110] lead to the synthesis of AVP in the hypothalamus, which is delivered to the posterior pituitary. From here it is released [111] to act on the kidney, resulting in renal water reabsorption, urinary concentration and water conservation [112]. The rise in pOsm stimulates thirst and an increase in water intake [111,113,114]. 

The osmotic set-point of hypothalamic osmoreceptor neurons prevents the pOsm from deviating by more than 1–2% in an individual [106,111], so that body hydration is maintained within narrow limits [110], which for plasma is between 285 and 295 mOsm/kg [2,9,10]. Thomas et al., cite a broader range for serum of 275 to <295 mOsmol/kg [115], but according to Stookey et al., <2% of individuals, consuming ≥3.0 L fluid per day, have a pOsm <285 mOsmol/kg [9].

This osmotic set point differs between individuals and is lower by 10 mmol/kg or more, than that which stimulates thirst [112]. This indicates that the signal to initiate water conservation via the kidney follows that to restore water-balance by increasing fluid intake. The AVP response to osmolar change is under genetic control [112,116]. 

### 4.2. Diagnosis of Systemic Dehydration

Clinical dehydration may be defined as a loss of body water, with or without salt, at a greater rate than its replacement [115]. Here we only consider “water-loss dehydration”, which is accompanied by plasma hyperosmolarity and intracellular dehydration [112]. It is also termed hypohydration, hyperosmotic hypovolaemia, and dehydration with minimal salt loss [112]. Plasma or serum osmolality, measured directly, or derived from its chemical composition [3,16] provides an index of body hydration for comparison with other clinical methods [2,107,117]. In acute studies, a loss of body mass ≥3%—signifying loss of TBW, recorded over a period of seven days, is also used as a reference standard in the detection of dehydration, [3]. Cheuvront, et al. consider that pOsm is the only useful static marker of dehydration, while pOsm, urinary specific gravity and body mass are valid dynamic markers [2].

Of the two recognised categories of dehydration, preclinical dehydration can be managed simply, by adjusting daily fluid intake, but current dehydration is life-threatening and demands urgent water replacement. Maughan has stated, “At the extreme, deprivation of water for more than a few days inevitably leads to death” [118]. Dehydration is a leading cause of hospitalisation and death in the elderly, from chronic diseases such as urolithiasis, hypertension, and coronary heart disease [119,120,121]. In the US NHANES III study, while the frequency of preclinical dehydration was reported to be 40% in those aged 70–90 years, a further 28% exhibited current dehydration [9].

A number of factors lead to a reduced water intake and a risk of greater water loss in the elderly. Older people have a smaller body fluid reserve than younger people, because they have a lower muscle volume [122,123]. They also lose more intracellular water in response to heat and exercise than the young [124]. Food intake and the frequency with which drinks are taken decrease with age [125] and the elderly fail to increase their fluid intake in response to dehydration [126]. This is in part due to a decreased sense of thirst [127] in which cognitive and physical factors may play a part [128,129]. As daily routines are lost and social contacts diminish, those with dementia may forget to drink [3] or fluid intake may be restricted deliberately, to control incontinence [3,130]. The urinary concentrating ability of the kidney also declines with age [3,128,131,132,133] and, additionally, an increased use of diuretics or laxatives in older people contributes to greater fluid loss [134]. 

The risk of dehydration is increased in elderly patients in long-term care. In the DRIE study, Hooper et al. [3] reported a frequency of 20% in a population of care home residents (n = 188) with a mean age 86 years, with renal, cognitive, and diabetic status consistently associated with the risk. Similarly, Wolff et al. [135] found a fivefold increase in the occurrence of dehydration in patients admitted to hospital from care homes, as opposed to, from home, with a roughly twofold greater risk of death in hospital [135].

This background emphasises the seriousness of dehydration as a source of clinical morbidity and the importance of detecting dehydration in the elderly, both in the wider community and particularly in individuals in care [136]. Dehydration is less likely to be overlooked in hospital populations, where blood samples are routinely taken and serum osmolarity can readily be calculated. Although it is accepted that pOsm or serum osmolarity provide the best single assessment of body hydration [3,122] such tests are not routinely available in the community or in residential care settings [137]. 

In such settings, assessment by health or social care workers is likely to be based on the findings of a reduced thirst, a sense of a dry mouth, furrowing of the tongue, slow capillary refilling of the nail bed, loss of skin turgor, a dry axilla, and increase in urine colour, which appear to be poor indicators of dehydration in older adults [3]. 

The report of Hooper et al. [138] and that of the earlier, US Panel on Dietary Reference Intakes [139] emphasised the need to develop a valid, simple and non-invasive screening test of dehydration in the community, to permit identification and management of water loss dehydration in older adults. 

### 4.3. Body Hydration and Tear Osmolarity

Although lacrimal secretion is influenced by vascular filtration pressure [140] it is the energy-requiring, secretory process that determines the final composition of the tears and hence its osmolarity [22,45]. Additionally, as Walsh and colleagues demonstrated, tOsm is influenced by whole body hydration [141,142,143]. 

In 2011, Fortes et al. exposed a group of normally hydrated young adults in a controlled environment chamber (CEC), to systemic dehydration equivalent to a 2–3% loss of body mass [141]. This was generated by a combination of physical exercise and water-deprivation. Their pre-exercise pOsm was 288 ± 5 mOsm/kg. As dehydration developed, tOsm followed pOsm closely and like the pOsm, was restored to normal during rehydration. In two trials, the mean tOsm correlated strongly with mean pOsm at each time point (r = 0.93, *p* < 0.001), suggesting that tOsm could serve as a surrogate for body hydration. Fortes et al., found that the sensitivity and specificity of tOsm as a test for systemic dehydration was, respectively, 80% and 92% [141].

**Figure 3:**
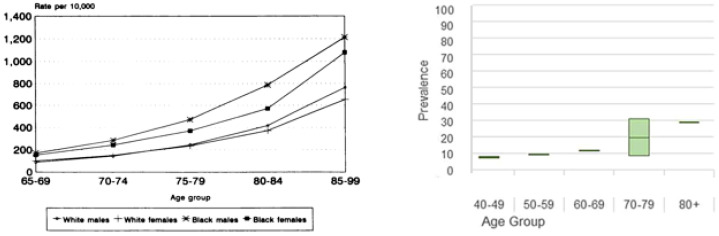
(**Left**) Rate of hospitalisation with any listed diagnosis of dehydration per 10,000 elderly Medicare beneficiaries, 1991. Reprinted with permission from ref. [5]. Copyright 2021 American Public Health Association. (**Right**). Prevalence of clinically diagnosed dry eye by age. Adapted with permission from ref. [152]. Copyright 2017 Elsevier Inc.

These studies were conducted in a population of young adult males and females. Importantly, as recognised by Walsh et al. [142], since the prevalence of both dry eye [144,145,146,147] and systemic dehydration [5,106], increases with age (Figure 3, the value of using a raised casual, or clinic-based tOsm in the diagnosis of systemic dehydration in the elderly would be reduced by the high risk of false positive results [148,149,150]. Tomlinson et al., emphasised at that time that the presence of tear hyperosmolarity (say >316 mOsm/L), whether due to systemic dehydration or to local eye disease, was in keeping with the diagnosis of dry eye whether consequent on systemic dehydration or a feature of local eye disease [142,151]. 

To overcome this difficulty we proposed that the task of diagnosing DED and systemic dehydration could be split into two, using a casual or clinic tOsm measurement for the diagnosis of DED and the tOsm measurement after a period of evaporative suppression, as the indicator of hydration state. The success of such an approach depends on the ability of suppression to bring down the tOsm to a basal level that is very close to the patient’s pOsm. This is discussed below.

## 5. The Concept of Basal Tear Osmolarity

### 5.1. Introduction

In a patient suspected of having dry eye disease, the presence of tear hyperosmolarity is based on a comparison of their tOsm with that from a large population sample of adults with normal eyes. Such a group, reported by Sullivan et al., included individuals of either sex, whose ages covered the range 18–82 years, (n = 299) [14]. It would be more valuable if that comparison could be made with that individual’s own tear osmolarity obtained before the onset of dry eye or better still with a basal tOsm reflecting the osmolarity of secreted tears in that individual, independent of environmental exposure. The former is not possible but the latter is.

We have hypothesised that it is possible to drive down tOsm to a basal level, close to that of the plasma, by a period of *total evaporative suppression* achieved either by *eye closure* or by *exposure to a relative humidity* of *100%* [11,12,13]. We have called this value the *basal tear osmolarity* (BTO). This basal value is thought to reflect the native osmolarity of the lacrimal fluid which, in the absence of tear evaporation and with adequate tear mixing and drainage, equilibrates with the interstitial fluid and plasma across the ocular surface epithelia. It is the level of tear osmolarity that must obtain when the eyes are closed during an overnight sleep. It is predicted that, because of this equilibration, and whether or not lacrimal fluid itself is truly iso-osmotic with plasma, its value will lie within the narrow range characteristic of the plasma, will be particular to an individual, will have a low variance on repeated testing, will always be lower than the tOsm measured in open-eye conditions and will not be significantly different from that of its fellow eye to which identical conditions apply. Considering eye closure as the basis of total evaporative suppression we may anticipate that exclusion of the tears from ambient environmental conditions will, with continued mixing and drainage of the tears, allow tOsm to fall to its basal level. The same will be anticipated for the hyperosmolar tears of a dry eye subject, although it would be predicted to take longer to reach the basal level. 

We have proposed that an individual’s BTO can be obtained by measuring their tOsm after a period of eye closure or by exposure of the subject in open-eye conditions to an RH of 100%. The former approach is offered as a potential clinical test and the latter as a means to explore the time course and dynamics of osmolar change during evaporative suppression. These approaches are described briefly below.

### 5.2. Measurement of Tear Osmolarity after Eye Closure

Some consideration is needed to select a duration of eye closure that will bring tOsm down to a stable BTO level. It is assumed that after a suitable period of time, depending on the rate of tear turnover, mixing and drainage, tears within the conjunctival sac will be completely replaced by the lacrimal fluid and a smaller amount of conjunctival and corneal fluid. Mixing will be enhanced by periodic eye movements. In patients with ADDE, reduced tear turnover could lengthen the time taken to reach the BTO, while increased epithelial permeability [148] would work in the opposite direction, by facilitating a faster ionic equilibration across the walls of the conjunctival sac. 

### 5.3. Estimating the Necessary Period of Eye Closure to Achieve the BTO

For the BTO to be accepted as a routine clinical test it will be important to keep eye closure time to a minimum so that the test is acceptable to the patient. A reasonable prediction of the necessary time, valid in both in patients with severe DED (and therefore high tOsm levels) and in subjects with normal tOsm values, may be inferred from the report of Zhu and Chauhan, who, on the basis of modelling the impact of raising the evaporation rate and then reducing it back to normal levels, predicted a restoration of tear osmolarity to baseline values in about 13 min [149]. Gaffney arrived at a similar figure when estimating the time required to bring down tear hyperosmolarity to a basal level in closed eye conditions, with complete suppression of evaporation [18]. 

With this background, 45 min of eye closure was adopted for the experiments described below. 

### 5.4. Measurement of Tear Osmolarity at High Ambient Humidity, in Open-Eye Conditions

Although exposure of an individual to an atmosphere 100% RH offers an alternative approach to the estimation of the BTO, eye closure is a more practical approach since it does not require a CEC or goggles designed to maintain a humid environment. Exposure to a humid environment offers some experimental advantages since the fall in tOsm can be tracked during exposure and tear mixing and drainage will be facilitated by spontaneous blinking. Both approaches allow the temporal aspects of osmolar recovery to be studied, as they were by Niimi et al. [69] (Figure 2).

### 5.5. Measuring Basal Tear Osmolarity after eye Closure or Exposure to High Ambient Humidity

We have reported preliminary studies to estimate the BTO in eight normal subjects and eight patients with mild dry eye, after periods of evaporative suppression achieved by either eye by closure or exposure to a high RH [12,13]. Recruitment criteria for normal subjects consisted of an Ocular Surface Disease Index (OSDI) score of <20, tOsm < 308 mOsm/L, Tear Break-Up Time (TBUT) >10 s, and corneal staining of <grade 2 using the Oxford scale [153]. The recruitment criteria for the DED participants included a tOsm of ≥308 mOsm/L [14], an OSDI score ≥20 [154], a TBUT of <10 s, and corneal staining of ≥grade 2. 

In the eye closure studies, closure was maintained for a period of 45 min and eye movements performed from time to time to achieve some degree of tear mixing. 

Concerning the high humidity studies, it is not possible to achieve an RH of 100% in a CEC, because of problems of condensation that occur with sustained use above 90% RH. Although the CEC at our disposal was only able to maintain an RH of 70% Madden et al., had considered this level to be sufficient to achieve significantly reduced evaporative suppression [87] and so these studies were conducted as planned, at 70% RH. Tear osmolarity was measured in both eyes every 15 min for a period 45 min, with the patient blinking spontaneously. 

Both studies were preceded by measurement of tOsm in clinic conditions outside the CEC to provide baseline values prior to evaporative suppression (Temperature ranged between 16 and 27 °C and Relative Humidity between 20 and 69%) The results of the studies are summarised here.

## 6. Inter-Eye Differences in Tear Osmolarity

A significant inter-eye difference was found in the DED group in *clinic conditions*: RE 295.9 ± 8.17 mOsm/L and LE 301.6 ± 10.63 mOsm/L (*p* = 0.017); but not in the normal group: RE 296.9 ± 6.86 mOsm/L and LE 297.8 ± 6.66 mOsm/L (*p* = 0.268). This is in keeping with other reports in DED [155].

Following eye closure—inter-eye tOsms were not found to be significantly different in either group, i.e., in the *normal group*: RE 286.3 ± 2.93 mOsm/L and LE 287.1 ± 4.02 mOsm/L (*p* = 0.383); and in the *DED group*: RE 285.5 ± 6.12 mOsm/L and LE 286.1 ± 6.60 mOsm/L (*p* = 0.809). 

After exposure to 70% RH—in the *normal group*: RE 295.3 ± 2.93 mOsm/L and LE 287.1 ± 4.02 mOsm/L (*p* = 0.383); in the *DED group*: RE 295.3 ± 10.58 mOsm/L and LE 291.0 ± 5.76 mOsm/L (*p* = 0.170).

## 7. Eye Closure Studies

In both normal subjects and dry eye patients, 45 min of eye closure significantly reduced tOsm in each eye to levels in the range accepted for effective plasma osmolality, i.e., between 285 and 295 mOsm/L. 

**Figure 4:**
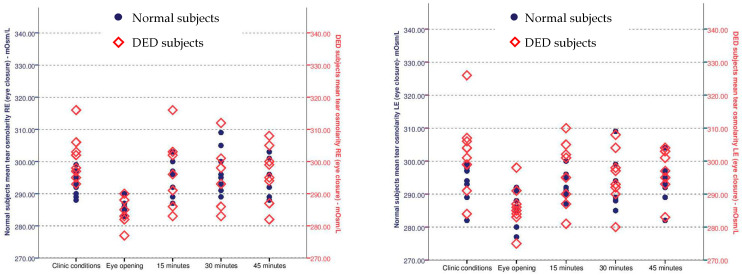
(**left**). Scatterplot displaying mean tOsm values for normal and Dry eye disease (DED) subjects RE after 45 min of eye closure and then every 15 min with eyes open at 45% relative humidity (RH). (**right**). Scatterplot displaying mean tOsm values for normal and DED subjects LE after 45 min of eye closure and then every 15 min with eyes open at 45% RH (Reprinted with permission from ref. [11]. Copyright 2021 Anglia Ruskin University ARU)

The average clinic tOsm in the RE of the eight normal subjects was 292.9 ± 3.91 mOsm/L, falling to 286.3 ± 2.71 mOsm/L following eye closure (*p* = 0.015). For the LE this was 293.1 ± 5.54 mOsm/L prior to eye closure and 285.9 ± 5.54 mOsm/L (*p* = 0.006) immediately after eye opening. Following eye opening the tOsms returned towards the clinic values in both eyes (RE *p* = 0.50; LE *p* = 0.51) in an atmosphere of 45% RH (Figure 4). 

In the DED group too, there was a significant decrease in tOsm in the RE and LE from that measured in clinic conditions. The average clinic tOsm in the RE was 301.3 ± 7.36 mOsm/L, falling to 283.8 ± 3.99 mOsm/L (*p* = 0.0002) (Figure 4 left). The average clinic tOsm in the LE was 302.3 ± 12.4 mOsm/L, falling to 286.1 ± 6.60 mOsm/L (*p* = 0.01) (Figure 4 right). It can be seen that although the criterion for recruitment into the dry eye group included a tOsm of ≥308 mOsm/L, at the time of study, the average tOsm was within the normal range (In different subjects, in the RE, values were 316 and 312 mOsm/L and in two other subjects, in the LE, the values were 312 and 326 mOsm/L). As with the normal group, tOsm returned towards the clinic value in both eyes (REP = 0.182; LEP = 1.0) on return to ambient clinic conditions 

## 8. High Humidity Studies

The fall in tOsm induced by exposure to high humidity was not as great as that achieved by eye closure, suggesting that evaporative suppression was incomplete at 70% RH. A significant fall in tOsm occurred in the normal group, in the LE only, but not in the dry eye group. The average clinic tOsm in the RE was 298.8 ± 8.91 mOsm/L, falling to 295.0 ± 5.50 mOsm/L (*p* = 1.108). In the LE the clinic value was 300.3 ± 7.48 mOsm/L, falling to 294.6 ± 4.31 mOsm/L (*p* = 0.045).

In the DED group there was a decrease in tOsm measured in the RE and LE from clinic conditions, following 45 min exposure to 70% RH, but neither eye reached significance. The RE started at 297.9 ± 7.01 mOsm/L and fell to 296.0 ± 9.47 mOsm/L (*p* = 0.294), while the LE started at 298.9 ± 7.14 mOsm/L and fell to 295.0 ± 14.7 mOsm/L (*p* = 0.081).

## 9. Discussion

### 9.1. Introduction

How does tear hyperosmolarity cause damage at the ocular surface?

Although the main focus of this article is the possibility of detecting systemic dehydration by measuring tOsm, it is also concerned with the diagnosis of DED and determining its severity. Tear hyperosmolarity is the key feature of DED, recognised to trigger all of the events that characterise it at the ocular surface, including the vicious circle of inflammation [77,78,79,80,156] that brings about an increase in epithelial permeability [148], and leads to epithelial cell death [83] and increased epithelial shedding [157]. Inflammatory events include the expression of inflammatory molecules [82] and invasion of the cornea and conjunctiva by inflammatory cells [81,82,150]. While the literature about these events is large, there are few articles on the manner by which tear and ocular surface tissue hyperosmolarity bring this about. One mechanism might be to raise the concentration of certain critical molecules in the tear which trigger a pathological pathway in the cells. A more evident one is through the presence of hypertonicity, leading to a relative dehydration and shrinkage of surface cells. This is important since it raises the question as to how hypertonic, tears of a given osmolarity are, and whether this is reflected in the TearLab^®^ measurement of osmolarity. The contribution of the urea molecule is a case in point.

The concentration of urea in the plasma (as BUN), is 7–20 mg/dL, equating with a molarity of 2.5–7.1 mOsm/L, so that, according to this argument, the VP and DFP values and the calculated serum values would exceed those by a device dependent on electrical conductivity, since they respond to the presence of urea and such devices do not (vide supra). Kang et al. [158] cited tear values from the literature of between 5 and 21 mOsm/L [96,159,160] and in his own studies, reported a mean value of 5.78 mMol/L which was 92.7% of the mean value in the blood. Thaysen [96] found the concentration of urea (T_U_) to be equal to that of the plasma (T_P_) and that the T_U_/T_P_ ratio was unchanged over a fourfold T_P_ range. 

The permeant behaviour of urea has a bearing on comparisons between tOsm measured by the TearLab device and pOsm, measured directly or calculated from a formula. If urea behaves as a permeant molecule at the ocular surface, and it does not do so at all sites [161], it will not contribute to the tonicity of the tears although its contribution to tear osmolarity will be accurately recorded by VP or DFP osmometry. This was the case in a group of 10 uraemic patients studied by Charlton et al. [155] where both tOsm and pOsm measured by DFP were raised, but despite tOsm levels of 347.2 ± 17.4 mOsm/L (range 312–375) there were no relevant signs of DED. The tears of these uraemic patients were hyperosmolar but probably not hypertonic. It may be predicted that the TearLab device would not register tear hyperosmolarity in these circumstances. It is relevant to the present article, where tOsm (the BTO) measured by the TearLab device is being compared with published figures for serum osmolality, that the formula used by Matz to derive the effective osmolality of the serum omits a component for urea, for the reasons given earlier, so it appears that the comparison is valid.

### 9.2. Exposure to High Ambient Humidity

In the personal studies described here, we attempted drive down tOsm either by means of eye closure or by exposing the patient to a high humidity in open-eye conditions. It was found that, despite the conclusions of Madden et al., 70% RH was insufficient to drive down tOsm significantly to the BTO [87]. Tear osmolarity was reduced in subjects with normal eyes and also those with mild DED but the average fall was only significant in the eye LE of the normal group. It appears that if high humidity is to be used to drive down tOsm to the BTO, it will be necessary to expose the eyes to an RH of 80–90% and the efficacy of this approach will need to be validated by parallel pOsm measurements. 

If successful, such studies will allow the dynamics of tOsm suppression to be determined, including the time taken to drive the tOsm down to its basal level. This will be a guide to the minimum period of eye closure necessary to induce a basal state, which is a requirement for devising a feasible clinical test. However, it can be a guide only, since the effect of each approach on tear dynamics differs in important ways. In open-eye, CEC conditions, tear flow and drainage are unrestricted and tear mixing is aided by spontaneous blinking and eye movements. With eye closure, the contribution of blinking and eye movements to mixing and drainage is lost and tear secretion is reduced [37,162].

### 9.3. Eye Closure Studies

In the eye-closure studies tOsm was driven down in both the normal eye and the DED groups to levels within the band reported for the plasma osmolarity. This would entitle these values to be termed BTOs. Because the tOsms of the DED patients were either raised only slightly or were in the normal range at the time of the study, the ability of eye closure to drive down a markedly raised tOsm to the BTO has not been tested. In published series of tOsm in patients with DED, upper figures for tOsm (two standard deviations above the reported mean) were 363 [163], 406 [164], 388 [165], 408 [51], and even 519 mOsm/L [56].

Future studies are needed in subjects with normal eyes and in patients with DED, in which tOsms measured after different periods of eye closure are directly compared with pOsms. It will be essential to include patients with severe DED and marked tear hyperosmolarity, in order to ensure that the tOsms can be driven down from a high level to a basal level in the time of the test. As noted earlier, predictions from modelling suggest that the eye closure time could be as short as 15 min [18,36].

The validity of the BTO concept was supported in another way in the eye-closure studies. 

In normal subjects, the mean inter-eye, tOsm difference was reported by Lemp et al. [84], to be 6.9 ± 5.9 mOsms/L, whereas in patients with mild or moderate DED the difference was 11.7 ± 10.9 mOsms/L and in those with severe DED, 26.5 ± 22.7 mOsms/L. In the studies we report here, whereas there was there was a significant inter-eye difference in pre-closure tOsm in the DED group but not the normal group in the eye-closure studies, there was no significant difference between the BTOs of the R or L eyes in either group. This is precisely what would be predicted if the BTO in each eye was determined by the same thing, i.e., the plasma osmolarity. 

### 9.4. Predicted Utility of the BTO in Estimating Systemic Hydration

A number of authors have demonstrated that eye closure, as in overnight sleep, causes a fall in tOsm to a low level, referred to as “hypo-osmolarity” by Niimi et al., resulting from the total suppression of tear evaporation [69]. We have proposed that since this tOsm level lies close to the pOsm it is appropriate to refer to it as the BTO, which could serve as a non-invasive measure of bodily hydration. We have demonstrated in preliminary studies that the BTO can be achieved with 45 min of eye closure and have suggested that this time could be further reduced, to create a point-of-care clinical test of bodily hydration that would be easy to conduct and acceptable to the patient.

The BTO is predicted to lie within the narrow limits dictated by the pOsm and therefore to be identical between the two eyes, independent of ambient clinic conditions or of the tOsm levels measured in the two eyes prior to the BTO measurement. It should not be modified by the tear hyperosmolarity of a dry eye patient. It is likely that the BTO, like the pOsm, is a signature value for any normally hydrated individual. 

Based on their studies in young adults, Fortes and colleagues originally proposed that measurement of the tOsm in open-eye, clinic conditions could provide “a practical and rapid hydration assessment technique” [141]. They subsequently pointed out [142,143] that there would be a risk of false positive results in elderly populations, due to the increasing incidence of both systemic dehydration [5] and of DED with age [152]. We have proposed that if the validity of the BTO concept is confirmed by direct comparison of the post-closure tOsms with the pOsms, use of the BTO could avoid this confounding factor.

Why should the BTO provide a better index of suboptimal hydration than the open-eye tOsm value? It is generally accepted that the tOsm measured casually or in clinic conditions deviates from that measured on waking, because the eyes are exposed to the effects of ambient air flow, humidity and temperature and to variations in psychic drive to the lacrimal glands. 

When dry eye tears are said to be “hyperosmolar” they are considered to be hyperosmolar with respect to normal tOsm values published in the literature [66]. It is this comparison that is used to separate DED into severity categories of mild, moderate, and severe [14]. However, it would be more appropriate to compare the patient’s raised tOsm with their own, basal, waking tOsm. We contend that this corresponds to the BTO and is very close to the pOsm in that individual. Since the pOsm is controlled within much narrower limits than the osmolarity of the tears in open-eye conditions and the osmolar set points concerned with regulating pOsm are under genetic control [112,116], it is likely that this pOsm band is individual to the subject. On the basis of this we suggest that the proper test of hyperosmolarity and hence measure of severity in a dry eye patient, is the difference between that patient’s *personal BTO* and *their casual or clinic-based tOsms*. New definitions of dry eye severity need to be worked out on this basis. In treating patients with dry eye and trying to restore tear osmolarity to normal, the BTO also provides an appropriate reference point against which to judge successful treatment. 

### 9.5. Tear Osmolarity at the Ocular Surface during Sleep

**Figure 5:**
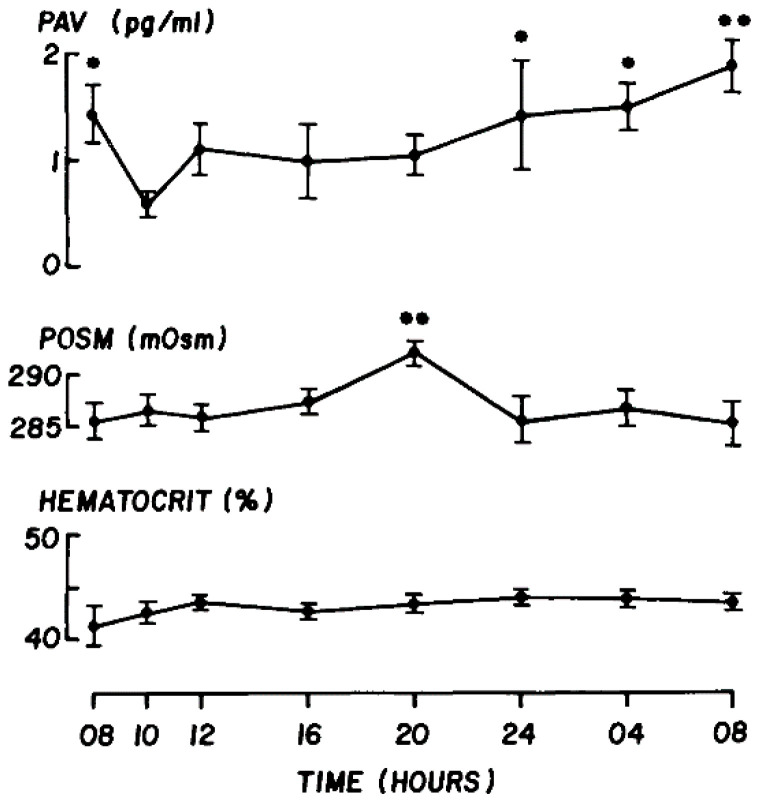
Mean values (±SE) of plasma arginine vasopressin (PAV), plasma osmolality, and hematocrit over 10 diurnal cycles, in 8 healthy males, age 21–28 years. * *p* < 0.05; ** *p* < 0.01. (Reprinted with permission from ref. [166]. Copyright 2021 Oxford University Press)

A central theme of this report is that in someone who is adequately hydrated, the simple act of eye closure, by preventing tear evaporation, drives tOsm down to a basal level close to that of the plasma. It is assumed that this is the case during overnight sleep, in subjects with normal eyes and those with dry eye. Thus, in DED the hyperosmolar state which perpetuates the vicious circle of inflammatory events at the ocular surface during the waking hours [77,78] must close down at night. It is intriguing that the inflammatory events of dry eye, despite this overnight protection from hyperosmolarity are perpetuated at the ocular surface by exposure to evaporation for the rest of the day. It may be assumed that in the normal eye during sleep, the complex, regulated, and contained inflammatory events in the conjunctival sac, referred to as “closed eye tears” [162], occur in conditions iso-osmotic with plasma and that this condition is also achieved in ADDE and EDE. This conclusion depends on the assumption that there is little systematic change in pOsm over the 24 h, but George et al. [166] studying the variation in plasma vasopressin in normal adult males, recorded an evening rise (8.0 p.m.) in pOsm of about 5 mOsmoles. Its basis was not explained—it was unassociated with a change in plasma sodium, potassium, or chloride (Figure 5).

### 9.6. Systemic Dehydration Dry Eye (SDDE)—A New Class of Dry Eye Disease

In 2012, Walsh et al., studied a population of 111 elderly adults (mean age 77 ± 8 years) admitted to an acute medical unit in Bangor, Wales, in whom open-eye tOsm, measured with the TearLab^®^ device, was compared with pOsm measured directly with a DFP device [143]. (Advanced Instruments. Model 330 MO). Dry eye was identified on the basis of tOsm alone (using the cut-off for mild/moderate DED of >316 mOsm/L or for severe DED, of >324 mOsm/L) or on a number of composite measures combining a tOsm cut-off with a DEQ-5 symptom measure or non-invasive breakup time measure. The osmolarity cut-off for the non-dry eye group was <308 mOsm/L.

In subjects not taking medications known to cause eye dryness, those classified as DED on the basis of osmolarity alone (with either cut-off) exhibited a significantly higher plasma osmolarity than non-DE subjects and there was a small but significant correlation between tOsm and pOsm and across the groups. The authors concluded that a proportion of those individuals diagnosed with dry eye had some degree of suboptimal whole-body hydration. They showed in a further pilot study (n = 8) that a programme of rehydration over 48 h resulted in a significant decrease in both plasma and tear osmolarity, with the pOsm changing from 296 ± 14 to 289 ± 13 mOsm/Kg and the tOsm from 335 ± 33 to 308 ± 10 mOsm/L. On this basis they suggested that a raised pOsm was sometimes the basis of an apparent DED and that it was particularly important to maintain an adequate level of hydration in elderly patients with DED, on the assumption that in a proportion of them their dry eye was secondary to systemic dehydration. 

Those subjects in whom both systemic dehydration and dry eye could be restored to normal by rehydration may be regarded as having a unique form of dry eye, different from ADDE or EDE, which we shall call here, Systemic Dehydration Dry Eye (SDDE). 

The distinctive characteristic of SDDE is that, because it is determined by a raised plasma osmolality, which will remain raised throughout the 24 h of the day in someone who has not been rehydrated, the tOsm should similarly remain raised throughout the 24 h including the hours of sleep. It may be hypothesised that this form of DED would evolve in two stages, the first involving a rise in BTO with a secondary rise in the casual tOsm level, still within normal limits and the second, in which a further rise in the BTO brings the tOsm into the DED range. A further consideration would be, how SDDE would interact with pre-existing ADDE or EDE. At the present time there have been no reports of the clinical features of this postulated form of DED in terms of the expression of inflammatory biomarkers at the ocular surface or its influence on closed eye tears.

### 9.7. A further Implication of the BTO—Apossible Correction Factor for Tear Biomarkers

Comparisons are frequently made between the concentrations of tear biomarkers in normal and dry eyes. A rise in the concentration of a biomarker in DED is taken to imply increased synthesis or reduced degradation of the biomarker. However, evaporative loss in DED will itself have a concentrating effect on less permeant molecules in the tears such as the biomarker proteins and the extent to which this has occurred may be gauged from the ratio of the casual, open-eye tOsm to the BTO. 

Applying this ratio as a correction factor could also give a clearer idea of the degree to which the secretion of lacrimal proteins (such as lysozyme and lactoferrin) are reduced in ADDE since their true reduction would be masked by the concentrating effect of dehydration [76].

## 10. Using Basal Tear Osmolarity as a Screening Test for Systemic Dehydration

### 10.1. In the Elderly

A number of authors have emphasised the need for a simple, rapid, and non-invasive test to diagnose systemic dehydration, particularly in the elderly [117,167,168,169,170]. The purpose of this report has been to suggest that measurement of the BTO, if its credentials are confirmed, could satisfy the role of a screening test for water-loss dehydration, in that, a positive result could lead to effective rehydration.

To pursue this, further studies are needed, as follows:

- A first requirement is to validate the concept of the BTO by showing that eye closure for 45 min does indeed achieve a basal tOsm level, close in osmolarity to an individual’s own pOsm and that this finding is highly repeatable in the individual. This requires contemporary measurements of pOsm and tOsm after 45 min of eye closure in participants with healthy eyes and a normal lacrimal function and in patients with DED (ADDE and EDE) showing a broad range of tear hyperosmolarities.
- High humidity CEC studies, at an RH 80–90%, are needed in patients showing a broad range of tear hyperosmolarity, mapping the time taken to bring down the tOsm to BTO level. These data could be used to design a shortened eye closure test acceptable for routine use in elderly patients. This would need to be trialed in eye closure studies.
- The shortened BTO test could then be trialed in a residential care-home setting, comparing BTO values with pOsm values on multiple occasions, to establish its credentials as a screening test for body-water deprivation. It would be important to measure pOsm directly, from blood samples and to estimate it from selected formulae.

The intention is to devise a point-of-care test that can be deployed at the bedside and used initially to establish the baseline hydration and dry eye status and then to monitor change over time. Considering an individual admitted to a care home, who has been confirmed to be well-hydrated on the basis of a blood test, the following tests would be carried out over time. On admission, a casual tOsm measurement would be made in each eye, followed by a bilateral BTO measurement. This would establish baselines for DED and hydration status. The bilateral, casual tOsm measurement would provide diagnostic DED information based on the presence of tear hyperosmolarity and evidence of a significant inter-eye difference in tOsm [84]. Since the right and left BTO values are not likely to be significantly different, further monitoring of hydration status would be based on unilateral BTO measurements carried out at agreed intervals or when the hydration status of the patient was called into question. The tOsm may be repeated bilaterally, with less frequency, to appraise DED status. Where the tOsm and BTO are performed on the same occasion the opportunity arises to diagnose several clinical states outlined in Table 2.

### 10.2. In Other Groups

While the focus of this report has been on dehydration in the elderly, dehydration is encountered at all ages [118]. There are many situations in which environmental conditions and physical exercise lead to or threaten to cause dehydration. The test described here could find a place in the community at large, in the study of body hydration in sports medicine, [170] and in field conditions in both sports medicine [169,171], and military environments [168]. 

Heat stress, induced by physical exercise or environmental conditions such as heat-waves, is an important factor precipitating hospital admissions in the elderly. As Maughan remarks, “All-cause mortality is increased when high temperatures persist for more than a few days” [118]. The EUROHEAT project, covering nine European cities, found that the impact of heat waves increased with age and was significantly greater among women aged 75–84 years in Mediterranean cities [118]. 

## 11. Conclusions

In summary, measurement of Basal Tear Osmolarity is proposed as the basis of a point-of-care screening test for preclinical and clinical dehydration. It has yet to be validated but if the concept is confirmed, the approach promises to be of particular utility in detecting dehydration in the elderly, who suffer a high morbidity and loss of life and also outside of hospitals and clinics, in the wider community across the age range. Studies are needed, to demonstrate that the BTO can act as a surrogate for pOsm, regardless of the starting tOsm level and within the period of a shorter test.

## Figures and Tables

**Table 1:** Average tear osmolarity (tOsm) values for normal subjects, from the literature.

Author	Method	n	Tear Osmolarity
Tomlinson et al., 2006 [66]	Meta-analysis: DFP and VP measurements. 1978–2005	815	302.0 ± 9.7 mOsm/L
Eldridge et al., 2010 [67]	TearLab^®^	30	301.8 ± 10.5 mOsms/L
Li et al., 2012 [68]	TearLab^®^	10	298.0 ± 14.2 mOsms/L
Niimi et al., 2013 [69]	TearLab^®^	38	297 ± 15 mOsms/L
Jacobi et al., 2011 [70]	TearLab^®^	133	301 mOsmol/L (range 298–304 mOsmol/L)
Sullivan et al., 2010 [14]	Tear Lab^®^	75	302.2 ± 8.3
Keech et al., 2013 [71]	Tear Lab^®^	10 15	304.0 ± 8.4 mOsm/L 301.2 ± 7.2 mOsm/L
Nolfi et al., 2017 [72]	Tear Lab^®^	20	295.4 ± 8.6 mOsm/L

Tear Osmolarity in Normal Eyes and in Dry Eye Disease. DFP = Depression of Freezing Point; VP = Vapour Pressure.

**Table 2:** Potential dry eye and hydration states.

Tear Osmolarity tOsm	Basal Tear Osmolarity BTO	Plasma Osmolarity pOsm	Clinical Status
T_1_	T_1_	P_1_	No DED; normal hydration
Up	T_1_	P_1_	ADDE or EDE; normal hydration
T_1_	Up	Up	Preclinical dehydration; tOsm not yet in the DED range
Up	Up	Up	Current clinical dehydration; SDDE Local dry eye tests may indicate there is coincident ADDE and EDE.

ADDE = aqueous-deficient dry eye; BTO = basal tear osmolarity; DED = dry eye disease; EDE = evaporative dry eye; pOsm = plasma osmolality or osmolarity; SDDE = systemic dehydration dry eye; T_1_ = normal tOsm; P_1_ = normal pOsm in euhydration; tOsm = tear osmolarity.
